# Evaluation of a moisturising micro-gel spray for prevention of cell dryness in oral mucosal cells: an *in vitro* study and evaluation in a clinical setting

**DOI:** 10.1111/j.1365-2354.2012.01349.x

**Published:** 2012-11

**Authors:** Y Ota, A Morito, K Fujisawa, M Nishida, H Hata, T Ueno, T Yurikusa, T Murata

**Affiliations:** Dental and Oral Surgery Division, Shizuoka Cancer Center HospitalShizuoka, Japan; Research and Development Department, Sunstar Inc.Osaka, Japan; Research and Development Department, Sunstar Suisse SAEtoy, Switzerland

**Keywords:** mucositis, stomatitis, dry mouth, cancer, micro-gel spray, moisturising product, oral mucosal cells

## Abstract

A moisturising micro-gel spray for prevention of dryness was compared with commercial products and artificial saliva *in vitro* and in a clinical setting in patients with cancer. Survival of cultured human gingival epithelial cells was evaluated after treatment with each product for 15 min. A dry test was performed for products giving a 50% survival rate, in which cell survival was measured after drying of cells treated with each product. The survival rates of cells treated with the micro-gel spray and artificial saliva were significantly higher than those of control cells. The micro-gel spray was then evaluated for 1 week in patients with symptoms of dry mouth caused by cancer treatment. There was significant improvement of these symptoms at night and on awakening and of subjective symptoms of decreased salivary volume (*P* < 0.05). Mean visual analogue scale scores also significantly decreased (*P* < 0.01). These data suggest that evaluation of moisturising products for dryness prevention can be performed in cultured cells, since products that performed well *in vitro* also showed good efficacy for symptoms of dry mouth. The micro-gel spray was particularly effective for relieving symptoms of dry mouth in patients with cancer.

## INTRODUCTION

Damage to the mucous membrane (mucositis) can occur either as a consequence of direct effects of the chemotherapy and the radiotherapy in head and neck cancer on the epithelial cells or as diminution of the protective effect of saliva (Epstein *et al*. 2002; Potting *et al*. 2006; Hong *et al*. 2009; Mencoboni *et al*. 2011). White discoloration of the mucous membranes mostly precedes the redness, oedema and lesions. Because of the pain, potential nutritional deficit and requirement for parenteral nutrition, significant reduction in the quality of life and the risk of mucosal infection and subsequent septicaemia, oral mucositis is an important clinical problem and needs a special attention especially in the case of malignancies (Harrold 2010).

Symptoms of dry mouth (xerostomia) occur in patients with severe diseases such as Sjögren syndrome and salivary gland disorders caused by radiation therapy for cancer, as well as with ageing, stress and drug administration. Under such conditions, the mucosa is continuously rubified, resulting in inflammation (Guggenheimer & Moore 2003; Wick 2007). Pilocarpine hydrochloride and cevimeline hydrochloride, which work as stimulators of muscarinic and cholinergic receptors on the exocrine surface, are used in many countries as prescription drugs for treatment of radiation-induced xerostomia (Chambers *et al*. 2007; Chitapanarux *et al*. 2008; Jha *et al*. 2009). However, these drugs have side effects such as gastrointestinal dysfunction and sweating that limit their use (Chambers *et al*. 2007; Berk 2008; Jha *et al*. 2009; Nakamura *et al*. 2009). Under these circumstances, alternative therapy is given for dry mouth, with use of moisturising gels, rinses and sprays, and in-hospital prescription drugs (Davies 2000; Dirix *et al*. 2007; Gil-Montoya *et al*. 2008; Hahnel *et al*. 2009).

A new method has recently been developed for evaluation of moisture retention based on the survival rate of dried oral mucosal cells as an objective marker for comparison of moisturising ingredients (Mori *et al*. 2010; Morito *et al*. 2011). We have used this approach to develop a new moisturising product, which we refer to as a micro-gel spray (Morito *et al*. 2011). Here, we compared the effects of the micro-gel spray on cell survival and dryness prevention *in vitro* with those of commercial moisturising products and artificial saliva. We then evaluated the performance of the micro-gel spray in a clinical setting in patients with symptoms of dry mouth caused by cancer treatment.

## MATERIALS AND METHODS

### *In vitro* evaluation

Human gingival squamous cancer-derived Ca9-22 cells were cultured with 10% foetal bovine serum in Eagle's Minimum Essential Medium (Sigma, USA) at 37°C under 5% CO_2_. The cells were cultured in a 96-well plate for 2 days until they became confluent. After removing the medium, the cells were washed with Phosphate Buffered Saline and treated at 37°C under 5% CO_2_ for 15 min with the moisturising products shown in Table 1. The test samples were then aspirated and the cells were washed with Phosphate Buffered Saline. The cell survival rates were measured in an Alamar Blue assay using a microplate spectrometer (Gemini XPS; Molecular Devices, San Jose, CA, USA) with absorption and excitation wavelengths set at 560 nm and 590 nm respectively. The survival rate was calculated relative to an assumed survival rate of 1 with Phosphate Buffered Saline, using the following formula: Cell survival rate = Fluorescence level of the treated group/Fluorescence level of the Phosphate Buffered Saline (control) group.

**Table 1:** Saliva substitute products studied *in vitro*

Product	Active and moisturising ingredients
Mouth wash A	Xanthan gum, polyvinylpyrollidone, carboxymethylcellulose
Mouth wash B	Lactoferrin, lysozyme, lactoperoxidase
Mouth wash C	Lactoprotein, lactoferrin, aloe vera
Mouth wash D	Hyaluronate
Mouth wash E	Xanthan gum, glycerine, hyaluronate
Mouth wash F	Polyglutaminate
Spray G	Glycerine, hyaluronate
Spray H	Sodium chloride, potassium chloride, calcium chloride hydrate, carmellose sodium
Micro-gel spray	Gellan gum, glycerine, glycosyltrehalose

Next, we evaluated the cell viability after drying for products with a relative cell survival rate of ≥50%. The cell culture and product treatment were performed as described above. After the 15-min culture, the medium and samples were completely aspirated, and the cells were kept at 20°C and humidity of 30% for 6 to 8 min (Sanyo, Osaka, Japan). Then, 200 µL of Phosphate Buffered Saline was added to each well and the cell survival rate was determined using the Alamar Blue assay described above.

### Subjects in evaluation of the micro-gel spray

The subjects were recruited from inpatients and outpatients with subjective symptoms of dry mouth after receiving head and neck radiotherapy and/or chemotherapy at Shizuoka Cancer Center from July to October 2008. The subjects were aged ≥20 years old. Patients prescribed drugs to improve salivary flow before the study and those who were determined to be inappropriate for participation in the study by the investigator due to a risk of aspiration were excluded. All patients received oral and written explanations of the objectives of the study, and written informed consent was obtained before participation in the study. This clinical evaluation was performed after review and approval by the ethical committee of Shizuoka Cancer Center.

### Baseline characteristics of the subjects

Age, gender, primary cancer site and symptoms of dry mouth (stimulated salivary volume and subjective symptoms of oral dryness) were recorded as baseline characteristics at the start of the study. Subjective symptoms of dry mouth and accompanying symptoms were determined using 13 items on a questionnaire that the subjects answered by selecting a score of 1 to 5 points (Table 2). Symptoms of dry mouth were also evaluated using a 100-mm visual analogue scale (VAS). Stimulated salivary volume was measured after chewing of gum for 5 min.

**Table 2:** Items on the questionnaire for subjective symptoms of dry mouth

Items on the dry mouth questionnaire	1	2	3	4	5
Do you usually feel dryness in the mouth?	Not at all	Seldom	Partial	Yes	Severely
Do you often drink water?	Seldom	Less	Partial	Often	Very often
Do you suffer from dry mouth at night or on awakening?	Not at all	Seldom	Partial	Yes	Severely
Do you have difficulty swallowing when you eat dry foods such as a cracker?	Not at all	Seldom	Partial	Yes	Very often
Do you feel oral dryness when you eat a meal?	Not at all	Seldom	Partial	Yes	Very often
Do you feel stickiness in the mouth?	Not at all	Seldom	Partial	Sticky	Very often
Do you feel that you have mouth odour?	Not at all	Seldom	Partial	Yes	Very often
Is speech difficult?	Not at all	Seldom	Partial	Yes	Very often
Do you have difficulty in swallowing food?	Not at all	Seldom	Partial	Yes	Very often
Do you often suffer from stomatitis?	Not at all	Seldom	Partial	Yes	Very often
Do you have difficulty in tasting?	Not at all	Seldom	Partial	Yes	Very often
Do you have a pain in your tongue or mouth?	Not at all	Seldom	Partial	Yes	Very often
Do you feel that you have less salivary volume?	Not at all	Seldom	Partial	Yes	Very often

### Evaluation method

The micro-gel spray was used as required for 1 week. Use of other products and drugs for prevention of oral dryness was prohibited during this period. Changes in subjective symptoms of dry mouth were evaluated based on an interview and changes in VAS scores obtained at baseline and after use of the gel spray for 1 week. Upon completion of the study, the subjects were asked about their general impressions of the micro-gel spray to evaluate its utility and effect duration.

### Statistical analysis

Differences in the *in vitro* cell survival rate were evaluated by anova and a Tukey multiple comparison test. Subjective symptoms of dry mouth before and after the trial were compared by Wilcoxon signed-rank test. For the 100-mm VAS scale, a *t*-test was performed to examine the difference between data at baseline and after use of the micro-gel spray for 1 week. All data were analyzed using spss ver.13 (IBM Inc., USA). A two-sided *P*-value <0.05 was considered to indicate a significant difference.

## RESULTS

### *In vitro* evaluation

**Figure 1:**
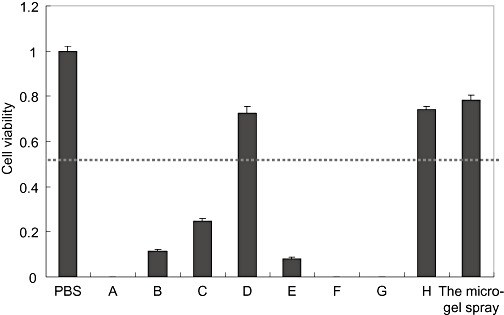
Relative cell viability after incubation with each moisturising product for 15 min. The viability of cells treated with Phosphate Buffered Saline (PBS) without drying was defined as 1. The mean ± SD of the cell viability relative to this value are shown for each product.

**Figure 2:**
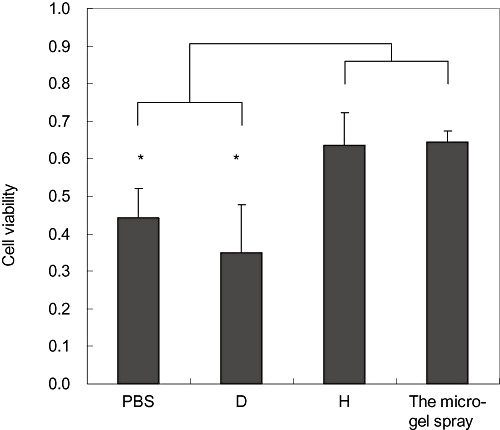
Relative cell viability after drying. After treatment with each product for 15 min, the cells were dried and cell viability was measured. The viability of cells treated with Phosphate Buffered Saline (PBS) without drying was defined as 1. The mean ± SD of the cell viability relative to this value are shown for each treatment. *P < 0.01 versus product H and Butler SG gel spray.

The survival rates after cells were immersed in individual moisturising products (saliva substitutes) for 15 min are shown in Figure 1. The cell survival rates with six commercial products were <50% compared with Phosphate Buffered Saline treatment (assuming a survival rate with Phosphate Buffered Saline of 1), and these products were excluded from further analysis. The other three products (two commercial products and the micro-gel spray) showed survival rates of ≥50% and were used in a drying test. The relative survival rates (Fig. 2) obtained after drying treatment for 6 to 8 min were 0.63 and 0.64 for product H and the micro-gel spray respectively. These rates were significantly higher than those with Phosphate Buffered Saline and product D (*P* < 0.01).

### Evaluation in a clinical setting

Twenty subjects began use of the micro-gel spray. However, one subject could not eat food and showed symptoms of taste disorder, and thus the product was discontinued. A second subject was excluded from the analysis due to incorrect answers in the evaluation. Therefore, the final analysis was performed for 18 subjects. The background factors at baseline in these subjects are shown in Table 3. The subjects were 11 male and seven female patients and their average age was 61.4 years old (range: 32 to 77 years old). The mean stimulated salivary volume over 5 min at the start of the study was 1.5 mL (range: 0 to 6.0 mL).

**Table 3:** Baseline characteristics in the 18 patients included in the analysis

Patient	Age	Gender	Tumour site	Treatment	Stimulated salivary volume (mL/5 min)
1	32	Female	Benign tongue angioma	Radiotherapy	1
2	58	Male	Oropharyngeal cancer	Chemoradiotherapy	2
3	57	Male	Unknown primary tumour	Chemoradiotherapy	6
4	73	Male	Neck lymphoma	Chemotherapy	1
5	54	Male	Hypopharyngeal cancer	Chemoradiotherapy	0.5
6	69	Female	Duodenal cancer	Chemotherapy	1
7	48	Female	Cancer of the tongue	Surgery	1
8	71	Male	Oropharyngeal cancer	Chemoradiotherapy	0.5
9	75	Male	Prostate cancer	Chemotherapy	<0.5
10	74	Female	Laryngeal cancer	Radiotherapy	5.5
11	59	Male	Epipharyngeal cancer	Chemoradiotherapy	0
12	60	Male	Oropharyngeal cancer	Surgery, radiotherapy	3.5
13	47	Female	Oropharyngeal cancer	Chemoradiotherapy	0
14	77	Male	Gastric cancer/cancer of the tongue	Surgery, chemoradiotherapy	1
15	66	Female	Laryngeal/hypopharyngeal cancer	Surgery, radiotherapy	1.5
16	59	Female	Breast cancer	Chemotherapy	1
17	63	Male	Hypopharyngeal cancer	Chemoradiotherapy	0
18	64	Male	Oesophageal/hypopharyngeal cancer	Chemoradiotherapy	0.5

**Figure 3:**
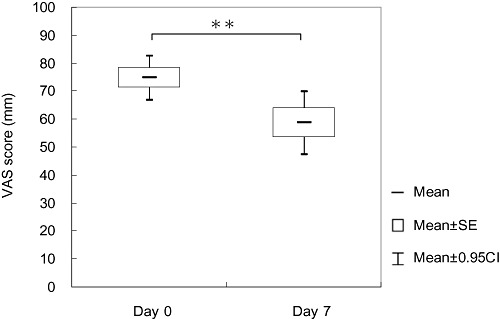
Box and whisker plots of mean visual analogue scale (VAS) scores on day 0 (baseline) and day 7 (at the end of the study). (*n* = 18) **P < 0.01 for day 0 versus day 7 by paired *t*-test.

The mean scores for subjective symptoms of dry mouth at baseline and on completion of the study are shown in Table 4. Significant improvement in dry mouth at night or on awakening and salivary flow occurred after 1 week of gel spray administration (*P* < 0.05). The mean VAS scores at baseline and at the end of the study are shown in Figure 3. The VAS score for oral dryness after treatment was significantly decreased by use of the micro-gel spray (*P* < 0.01 by *t*-test).

**Table 4:** Mean subjective dry mouth scores on day 0 (baseline) and day 7 (at the end of the study) in patients who received gel spray treatment (*n* = 18)

Subjective symptoms of dry mouth	Day 0 (SD)	Day 7 (SD)	*P*-value
Dryness in the oral cavity	4.11 (0.90)	3.67 (0.97)	0.054
Fluid intake	3.78 (1.26)	3.33 (1.46)	0.054
Dry mouth at night or on awakening	4.28 (0.90)	3.72 (1.07)	0.026*
Difficulty of swallowing dry food	4.11 (1.08)	3.72 (1.07)	0.319
Feeling of oral dryness during eating	3.94 (1.11)	3.39 (1.15)	0.133
Sticky feeling in the oral cavity	3.67 (1.14)	3.50 (1.25)	0.408
Subjective feeling of mouth odour	2.22 (0.94)	2.22 (1.22)	0.951
Difficulty with speech	3.50 (0.86)	3.56 (0.98)	0.725
Difficulty swallowing	3.61 (1.15)	3.50 (1.10)	0.914
Stomatitis	2.39 (1.20)	2.39 (1.15)	1.000
Difficulty with tasting	3.17 (1.15)	2.94 (1.16)	0.248
Pain in tongue and oral cavity	2.44 (0.78)	2.44 (1.10)	1.000
Awareness of having less salivary flow	4.39 (0.70)	3.89 (1.08)	0.041*

Smaller numbers (closer to 1) indicate a less severe symptom.

* *P* < 0.05 versus mean score on day 0 by Wilcoxon signed-rank test.

The results of evaluation of the micro-gel spray on a 5-point scale are shown in Table 5. Single use of the micro-gel spray resulted in a mean effect duration of 30 to 60 min. The scores for ease and pleasance of use, mucosal sensitivity, refreshment, change of dry mouth feeling and willingness to continue use were 3.9, 3.7, 3.5, 4.1 and 4.1 respectively.

**Table 5:** Overall impression of the micro-gel spray (*n* = 18 patients)

	Mean	Median	Mode	Range
Duration of effect	–	0.5–1 h	0.5–1 h	<0.5–2to3 h
Ease and pleasance of use	3.9	4	4	1–5
Mucosal sensitivity	3.7	4	4	2–5
Refreshment	3.6	4	4	2–5
Change of dry mouth feeling	4.1	4	4	2–5
Willingness to continue use	4.1	4	5	2–5

Based on a 5-point scale, with 5 being the most positive score and 1 the least positive score.

## DISCUSSION

The micro-gel spray was developed as a moisturising product for dry mouth caused by cancer treatment. In this study, the effects and utility of this product were evaluated. The results showed that the gel spray increased cell survival under dry conditions *in vitro*. In contrast, treatment of cells with six of eight commercial products resulted in cell survival rates of <50% after 15-min immersion and some products caused alteration of cellular morphology in this time period. This suggests that these products might cause direct damage to the cells.

Many products aimed at moisture retention and moistening have been developed for treatment of patients with symptoms of dry mouth and several of these products physically coat oral tissues. These moisturising products often contain carboxymethyl cellulose as a thickener and mucin and xanthan gum as moisturising and viscoelastic materials respectively. The micro-gel spray contains glycerine, gellan gum and glycosyltrehalose, which were selected by screening for ingredients with protective effects on cells (Morito *et al*. 2011). Trehalose has been shown to protect corneal epithelial cells against death by drying (Matsuo 2007). These effects may be caused by the trehalose sugar stabilising lipids and proteins on the cell membrane under conditions of oral dryness (Crowe *et al*. 1987).

The micro-gel spray significantly improved symptoms of dry mouth at night and on awakening and reduced salivary flow in patients with dry mouth caused by cancer treatment. A significant improvement in VAS scores for a post-treatment feeling of oral dryness was also obtained. The VAS score has been widely used for evaluation of symptoms of dry mouth (Shahdad *et al*. 2005; Dirix *et al*. 2007). In the current study, improved VAS scores corresponded with the results of a questionnaire survey, in which approximately 90% of the subjects answered that their symptoms of dry mouth were improved. In contrast, there was no improvement in swallowing of dry food, feeling of oral dryness during eating, sticky feeling in the oral cavity, subjective feeling of mouth odour, difficultywith speech and swallowing, development of stomatitis, difficulty with tasting, and pain in the tongue and oral cavity after use of the micro-gel spray. In a study of Aloe Vera Gel, carboxymethyl cellulose spray, porcine stomach mucin spray and rapeseed oil, symptoms of dry mouth after radiotherapy were also improved, but there were no significant improvements in difficulty with swallowing during eating, the need for water drinking, and difficulty with tasting (Momm *et al*. 2005).

One of the 20 subjects discontinued treatment with the micro-gel spray on the third day of use due to a bad taste. However, this subject might have had a taste disorder due to coated tongue due to the absence of oral food intake in the terminal phase. Approximately 80% of the subjects who continuously used the micro-gel spray felt that the usability was good or better, and only one subject felt that it was bad. The mean stimulated salivary volume in 5 min at the start of the study (baseline) was very low (1.5 mL), and thus the subjects had severe dry mouth. Oral symptoms caused by cancer treatment include oral mucositis, dentin hypersensitivity and oral dysgeusia, in addition to dry mouth (Fisher *et al*. 2003). Patients with these conditions require mild salivary substitute products with reduced sweetness (artificial saliva). Approximately 80% of the subjects were satisfied with the usability of the micro-gel spray, which suggests that the product has high tolerability for patients with oral symptoms. The median effect duration after single use of the micro-gel spray was 30 to 60 min, and the longest duration was 2 to 3 h. The effect duration of commercial moisturising products has also been reported to be about 30 to 60 min, although this depends on individual patients and symptoms (Regelink *et al*. 1998).

A more detailed evaluation of the effects of the micro-gel spray on dry mouth will require a clinical trial with a control group and a greater number of subjects. However, the product had a protective action against cell death in dry oral mucosal cells, and improved symptoms of dry mouth and showed high tolerability in patients receiving cancer treatment. The consistency of the *in vitro* and clinical results also suggests that the cell evaluation method is useful for assessment of moisturising products.
